# Early access provision: Awareness, educational needs and opportunities to improve oncology patients’ access to care

**DOI:** 10.3389/fonc.2022.714516

**Published:** 2022-10-26

**Authors:** Andriy Krendyukov, Sanjay Singhvi, Yianick Green-Morrison, Markus Zabransky

**Affiliations:** ^1^ Andriy Krendyukov VP Medical Affairs, Apogenix AG, Heidelberg, Germany; ^2^ Sanjay Singhvi/Yianick Green-Morrison VMLY&R Health, London, United Kingdom; ^3^ Markus Zabransky, Global Medical Affairs, Sandoz GmbH, Holzkirchen, Germany

**Keywords:** early access provision, innovative medicinal product, oncology, access to care, education, COVID19

## Abstract

**Background:**

An unmet medical need exists for many oncology patients who cannot be treated satisfactorily by available therapeutic options. Early access provision (EAP) is endorsed by competent authorities to improve patient access to innovative medicinal products (InMPs). This paper determined awareness and understanding among practicing physicians of integrated EAP protocols, and of the procedures involved in EAP applications for oncology trials prior to marketing authorization.

**Methods:**

An on-line, fully anonymous survey reaching out to more than 3,258 physicians (including practicing oncologists) was initiated between November 2020 - January 2021. Participants were questioned about their knowledge and understanding of EAP and the decision processes involved, level of experience, interest for further educational activities and opportunities to improve the process, both in general and specifically during the COVID-19 pandemic. The frequency of EAP protocols for oncology InMPs was identified by a search of ClinicalTrials.gov and EU Clinical Trials registers.

**Results:**

Survey results (75% oncologists) indicated 75% of respondents were ‘very comfortable’ or ‘comfortable’ with using EAP for their patients, but only 54.5% correctly answered the specific knowledge-based question related to the EAP definition. For 56% of respondents, experience with EAP in daily practice was very limited. Two-thirds indicated an average or lower level of understanding about the application process and regulatory requirements involved (65.2% and 66.0%, respectively). Knowledge on data collection and serious adverse event reporting under EAP was lower at 57.8% and 50.5% of respondents, respectively. Awareness of physician responsibilities was high in 59.7% of respondents, but fewer understood roles and responsibilities of manufacturing companies (31.2%). Most indicated they would consider clinical efficacy and safety data from comparative phase III randomized controlled trials as of high importance to support their decision to apply for EAP (93.4% and 86.8%, respectively). During the COVID-19 pandemic, the majority of respondents highlighted the need to improve and adapt EAP with regard to the application process and documentation (83.8%), InMP supply and logistics (88.4), and safety reporting process (78.0%). Of identified oncology trials with a ClinicalTrials.gov protocol, only 149 (0.4%) included EAP, and 23 used the data to receive a marketing authorization during the period Jan 2015 to December 2020. Of oncology trials with a EudraCT protocol, only 21 (0.23%) included EAP, of which 6 were used to receive a conditional or full marketing authorisation over the same period.

**Conclusion:**

Use of EAP in daily practice remains limited. Challenges posed by the EAP process, together with a lack of education on this topic, might contribute to its under-utilization and influence access of oncology patients to care. Continuous educational efforts from different stakeholders are required to better inform and support practicing oncologists during the EAP application process and regulatory framework follow up. Education should also be provided on EAP roles and responsibilities, monitoring, and potential adaptations when faced with specific challenges, such as the current COVID-19 pandemic.

## Introduction

The provision of early access to innovative therapies with added value compared to available alternatives is essential where there is an unmet clinical need in terms of adequacy of available treatments and disease severity or burden. This is often the case in oncology, and particularly in rare cancers. Globally, there were an estimated 19.3 million new cancer cases and almost 10.0 million cancer deaths in 2020 (1). Rare cancers are estimated to account for around 20-24% of all cancers diagnosed, albeit with disparities in both incidence and survival between different countries (2–4). However, clinical trials of innovative medicinal products (InMPs) in oncology and particularly for rare cancers are subject to a number of inherent challenges (5, 6). Foremost, the patient population available is generally made up of those who have not responded to all existing standard therapies, for who there remain very limited treatment options, and who may have a short life expectancy. The difficulty of conducting trials in these populations has been recognized by the US Food and Drug Administration (FDA) and European Medicines Agency (EMA), who have made substantial efforts to expedite review and approval with the introduction of breakthrough therapy designation (7) and priority medicine designation (8), respectively. However, even in the event of a positive competent authorities’ decision, there may still be a lengthy delay before oncology patients can gain access to new and innovative therapies. Added to this there are also issues related to disparity in cancer care and opportunities to participate in clinical trials. For example, for patients from certain racial or ethnic backgrounds, for those living in rural areas, or for those with lower income and education levels (9). All populations should have an equal opportunity to benefit from cancer prevention, early detection, and available treatments, including InMPs.

Patient recruitment issues are one of a number of factors driving both the length and cost of rare disease trials, and delaying patient access to InMPs (5, 10). When patients are not eligible or unable to participate in clinical trials, and have exhausted all available treatment options for an immediately life-threatening or serious disease, one avenue to gain access to an InMP for treatment is *via* Early Access Provision (EAP). This is known as expanded access in the USA, and individual named patient or compassionate use in the EU. These pathways are intended to provide more rapid access to potentially life-lengthening or life-saving treatments whose approval might otherwise come too late. They also go some way to meeting the primary ethical argument for earlier access, which is that patients should have a right to alleviate extreme suffering and to decide their own risk-benefit thresholds for a potentially life-lengthening or life-saving InMP (11).

Similar schemes are also operative in other global jurisdictions albeit with varying regulations (12, 13), including Japan (Expanded Trial) (14), Canada (Special Access Program) (15), Australia (Special Access Scheme) (16), and South Korea (Treatment Use of an Investigational New Drug) (17). The programs regarded as most well established are those in the US and Europe (6), and these will be the focus of this article. In the US, “Expanded Access” can be granted for three types of investigational new drug (IND) use (1): individual patient IND use (2); limited use for an intermediate-size IND patient population; and (3) treatment IND for widespread use under a treatment protocol; the latter might occur after a successful trial of an experimental agent has been concluded but before it has received FDA approval (18, 19). In Europe, the Committee for Medicinal Products for Human Use (CHMP) recognizes two types of IND use: those applicable for a cohort of patients known as “Compassionate Use” and those for “Named Patient Use” (20). Strict conditions apply for InMPs that have not yet been approved as the investigational agent may, or may not, be effective for the intended indication, and its use may cause unexpected serious side effects. At a minimum, it must be demonstrated that the condition is serious or immediately life-threatening, that there are no similar or satisfactory alternative therapies, and that access will not interfere with pivotal clinical trials (11). In addition, any potential patient benefit must also justify the potential risks. For individual patients, this might only require a physician to conclude that the risk posed by the InMP is no greater than the disease itself. However, the FDA must find sufficient evidence of safety and effectiveness before it grants an expanded-access IND protocol involving large numbers of patients with serious disease.

Recently reported data from the US Center for Drug Evaluation and Research (CDER) and Center for Biologics Evaluation and Research (CBER) revealed that applications for expanded access pathways dealing with patient cohorts remain low, despite high approval rates (6, 21). The aim of this perspectives article was to survey practicing physicians to determine the challenges they face when dealing with EAP and to identify potential educational needs that could improve access of patients with rare diseases, including cancers, to InMPs. This was supplemented with a search of the ClinicalTrials.gov and EU Clinical Trials registers to investigate the frequency of oncology InMPs that had applied for EAP prior to marketing authorization.

## Understanding, knowledge gaps and acceptance of EAP among practicing oncologists − results of an online survey

### Survey aims and methodology

To further explore the understanding, knowledge gaps and acceptance of EAP pathways a fully anonymous on-line survey was conducted between November 2020 and January 2021. The survey platform was hosted by Alchemer (formerly SurveyGizmo), an application that allows individuals and organisations to create, run, distribute surveys, and then analyze the results. The survey was created in the collaboration with System Analytic. All survey components were password protected. Response bias was limited by offering no financial incentive for completing the survey. In addition, each participant was followed up three times (once per week) in case of no response, after which they were marked as ‘Do not follow up.’ In each follow-up e-mail there was an ‘unsubscribe’ option if the person did not want to be contacted again. A total of 3258 physicians (including practicing oncologists) were questioned about their knowledge and understanding of EAP, their level of knowledge and experience with the process, understanding of their own responsibilities, and their interest in further educational activities and opportunities to improve the process. Attributes were rated on a 5-point scale from 1 not important/strongly disagree/very low, to 5 very important/strongly agree/very high. Only fully completed responses were included in the analysis of which 148 were returned.

### Survey results

The majority of respondents were located in Europe and North America (50% and 23%, respectively), with additional representations from South-East Asia, China, Japan, and Australia (**Supplementary Figures 1A, B**). Most of the responders were oncologists (75%), but other specialties were also represented including pulmonary, hematology, and cardiology.

Although 75% of respondents reported they were ‘very comfortable’ or ‘comfortable’ with the concept of using EAP for their patients, only 54.5% correctly answered the specific knowledge-based question related to the EAP definition. Over half the respondents (56%) had limited experience with EAP in daily practice, with requests of ≤2 patients/year; only 19% handled more than five requests per year. When questioned about their knowledge of EAP, around two-thirds indicated an average or a lower level of clear understanding about the application process and regulatory requirements involved (65.2% and 66.0%, respectively). For data collection and serious adverse event reporting under EAP, 57.8% and 50.5% of respondents, respectively, had an average or lower level of understanding. Awareness about the roles and responsibilities of the physician was high to very high in 59.7% of respondents. In contrast, the majority had a poor understanding of the roles and responsibilities of manufacturing companies, and patients and their families, 68.8% and 63.3%, respectively. A high proportion of respondents reported that they would consider the availability of clinical efficacy and safety data from comparative phase III randomized controlled trials as of high importance to support their decision to apply for EAP (93.4% and 86.8%, respectively); other evidence and study types were rated substantially lower (e.g. pharmacokinetic/pharmacodynamic data [22%], mode of action data [47.3%], and preclinical safety data [52.8%]). Responses of respondents from Europe and the US were similar in terms of their ratings on the importance of different types of efficacy and safety data to support EAP applications (**Supplementary Figures 2A, B**).

### Implications of survey findings

During the current COVID-19 pandemic, the majority of respondents highlighted the need to improve and substantially adapt EAP with regard to the application process and documentation (83.8%), InMP supply and logistics (88.4), and safety reporting process (78.0%).

**Figure 1:**
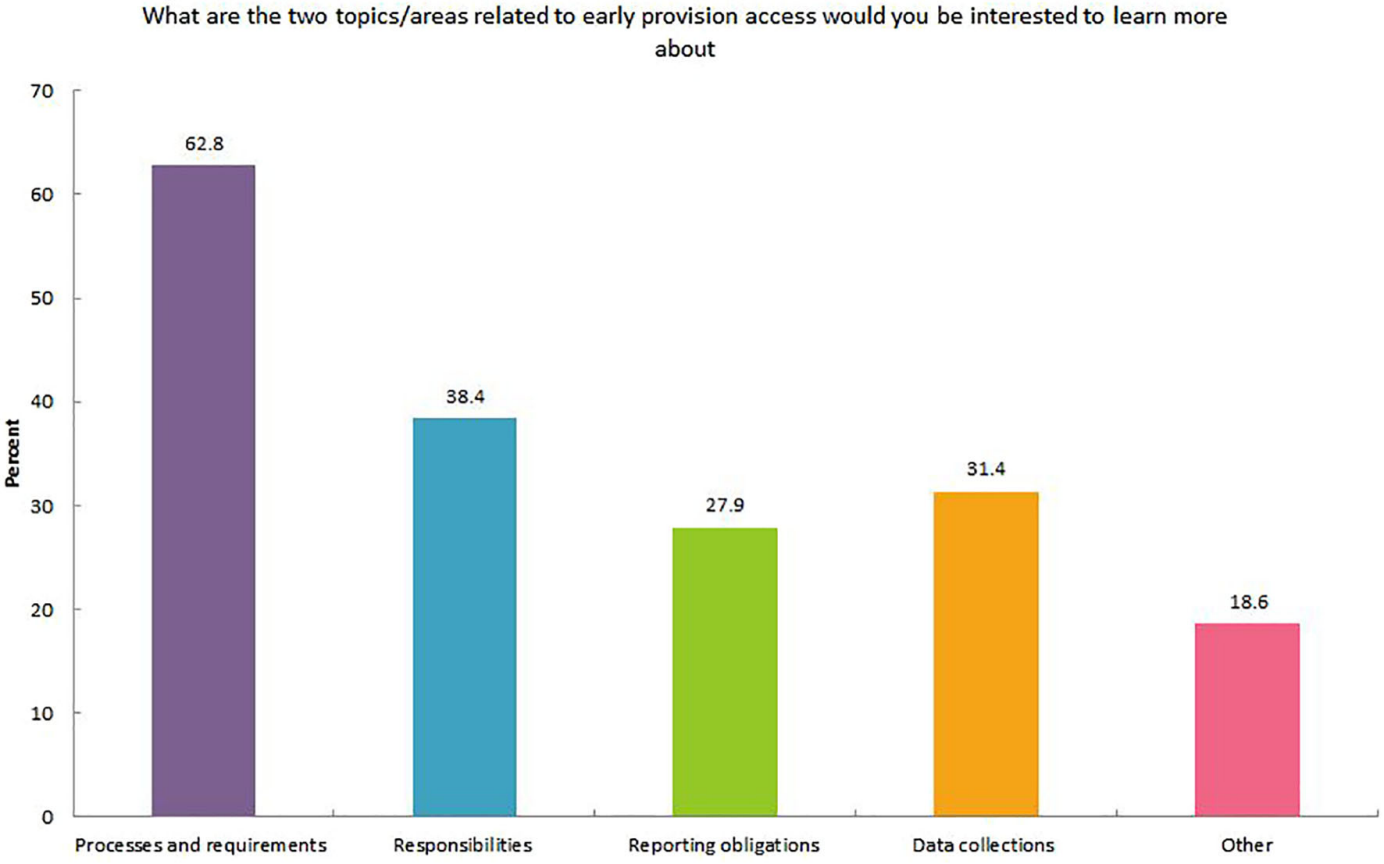
Early access provision knowledge improvements requested by healthcare providers.

A need for further education on the processes and regulatory requirements, precise/defined roles and responsibilities, and data collection involved in EAP was identified by 62.8%, 33.2%, and 31.4% of respondents, respectively (**Figure 1**). A number of common challenges when dealing with EAP were identified. These included: time constraints related to the paperwork involved in the application and complying with the responsibilities associated with using the treatment if access granted (76.7%); organizational roles and responsibilities (46.5%); lack of information (43.0%); and financial queries (31.4%) (**Supplementary Figure 3**). Responses of respondents from Europe and the US were similar in terms of the challenges faced during EAP applications (**Supplementary Figure 4**).

## Oncology InMPS with early access provision prior to marketing authorization − search of clinical trial databases

To determine how many cancer products had associated patient group applications for EAP prior to marketing authorization a search of the ClinicalTrials.gov and EU Clinical Trials registers was performed for the period 1 Jan 2015 to December 2020. This search period was selected to capture the current situation with regard to trials with integrated EAP protocols. In particular, the ClinicalTrials.gov register is optimized for such a search incorporating an option in its advanced search applied filters to select expanded access and to choose from three different expanded access protocol types: individual patient, intermediate-size population, and treatment IND/protocol.

The search identified 38,407 cancer trials with a US location, of which only 149 (0.4%) offered EAP. Of these, clinical data from 23 were used to receive a marketing authorization during the period 1 Jan 2015 to December 2020 (**Table 1**). Similarly, a search of the EU Clinical Trials Register revealed that of the 8,981 cancer trials listed with a EudraCT protocol, only 21 (0.23%) included EAP. Of these, clinical data from six studies were used to receive a conditional or full marketing authorisation during the same period (**Table 1**). It should be noted that the majority of studies with integrated EAP were to allow patients who had participated in a clinical trial to continue with the treatment and bridge the gap between trial completion and marketing authorization. For example, the osimertinib EAP was developed to provide compassionate access to osimertinib in the USA after enrolment in clinical trials had closed, but prior to FDA approval for patients with advanced or metastatic EGFR* *T790M-positive non-small cell lung cancer following progression on prior EGFR-TKI therapy (22). Trials with integrated EAP are clearly described as expanded access in the ClinicalTrials.gov register, but frequently only referred to as open-label extension studies in the EU register (12). EAP may also be granted in countries without access to the InMP outside of a clinical trial, although the agent may be approved in other regions. Such was the case for ruxolitinib, the first Janus Kinase (JAK) inhibitor approved for the treatment of myelofibrosis (23). The JAK Inhibitor RUxolitinib in Myelofibrosis Patients (JUMP) study, a phase IIIb trial with integrated EAP, has become the largest clinical trial in patients with myelofibrosis treated with ruxolitinib to date, with patients enrolled and treated in a setting similar to routine clinical practice (23).

**Table 1:** Oncology investigational new drugs with an expanded access protocol prior to marketing authorization by the FDA or EMA for the period 2015-2020.

Year	Trial identifier	InMP/INN	Date of approval	Description
Drugs with FDA marketing authorization				
**2020**	NCT04377152	Gallium 68 PSMA-11	12/1/2020	Expanded access 68Gallium-PSMA-11 PET for prostate cancer
**2019**
**2018**	NCT03070093	Gilteritinib	11/28/2018	Expanded access study in patients with FMS-Like Tyrosine Kinase 3 (FLT3) mutated or relapsed acute myeloid leukemia
	NCT03025360	Larotrectinib	11/26/2018	Expanded access for treatment of cancers with a NTRK gene fusion (biomarker)
	NCT03245424	Ivosidenib	7/20/2018	Expanded access program in relapsed/refractory acute myeloid leukemia with an IDH1 mutation
	NCT03523338	Apalutamide	2/14/2018	Expanded access protocol for participants with non-metastatic castration-resistant prostate cancer
	NCT02705313	Lutetium Lu 177 dotatate	1/26/2018	Expanded access protocol for patients suffering from inoperable somatostatin receptor positive neuroendocrine tumors
**2017**	NCT02792725	Abemaciclib	9/28/2017	Expanded access program for patients with metastatic breast cancer
	NCT02792725	Abemaciclib	9/28/2017	Expanded access program for patients with metastatic breast cancer
	NCT02624570	Midostaurin	4/28/2017	Expanded access program for newly diagnosed FLT3 mutated acute myeloid leukemia patients
	NCT03025867	Niraparib	3/27/2017	Expanded access protocol for patients with recurrent ovarian cancer
	NCT03089658	Avelumab	3/23/2017	Expanded access protocol for treatment of metastatic Merkel cell carcinoma
**2016**	NCT03994627	Olaratumab	10/19/2016	Expanded access program for patients with soft tissue carcinoma
	NCT02589717	Atezolizumab	5/18/2016	Expanded access study in participants with locally advanced or metastatic urothelial carcinoma after failure with platinum-containing chemotherapy
	NCT03123029	Venetoclax	4/11/2016	Expanded access protocol for chronic lymphocytic leukemia in patients with a specific chromosomal abnormality
**2015**	NCT02271139	Alectinib	12/11/2015	Expanded access study for patients with anaplastic lymphoma kinase (ALK)- rearranged non-small cell lung cancer after disease progression
	NCT02368301	Elotuzumab	11/30/2015	Expanded access protocol to provide elotuzumab in combination with lenalidomide and dexamethasone in patients with relapsed or refractory multiple myeloma
	NCT02477891	Daratumumab	11/16/2015	Early access treatment for relapsed or refractory multiple myeloma
	NCT02451852	Osimertinib	11/13/2015	Expanded access protocol for patients with advanced/metastatic epidermal growth factor receptor T790M mutation-positive non-small cell lung cancer
	NCT00210665	Trabectedin	10/23/2015	Expanded access protocol for participants with locally advanced or metastatic soft tissue sarcoma who have persistent or recurrent disease
	NCT02286492	Trifluridine and tipiracil	9/22/2015	Expanded access protocol for patients with metastatic colorectal cancer
	NCT02568943	Panobinostat	2/23/2015	Expanded access protocol of Panobinostat in combination with bortezomib and dexamethasone for relapsed and refractory multiple myeloma
	NCT02211222	Lenvatinib	2/13/2015	Expanded access program for the treatment of radioiodine-refractory differentiated thyroid cancer
	NCT02142868	Palbociclib	2/3/2015	Expanded access study in combination with letrozole for HR-positive, Her2-Negative advanced breast cancer
**Drugs with EMA marketing authorization (EudraCT Number)**				
**2020**
**2019**
**2018**	2018-001321-68	Gemtuzumab ozogamicin	4/19/2018	Expanded access protocol for treatment of patients In the United States with relapsed/refractory acute myelogenous leukemia
**2017**
**2016**
**2015**	2014-001700-21	Blinatumomab	11/23/2015	Pediatric and adolescent subjects with relapsed and/or refractory B-precursor acute lymphoblastic leukemia
	2014-003239-21	Panobinostat	8/28/2015	An open-label, multi-center, expanded treatment protocol of oral panobinostat in combination with bortezomib and dexamethasone in patients with relapsed and relapsed and refractory multiple myeloma
	2014-002834-30	Talimogene Laherparepvec	12/16/2015	A Phase 3b, multicenter, open-label, single-arm, expanded access protocol of talimogene laherparepvec for the treatment of subjects in Europe with unresected stage IIIB to IVM1c melanoma

## Discussion

Our findings indicate that use of EAP is limited in general, with a search of clinical trials databases identifying only a few clinical trials with integrated EAP protocols. This is despite the fact that regulatory bodies approve the majority of the requests they receive (24). The high proportion of regulatory body approval for EAP suggests that in most cases it is the manufacturing companies who are reluctant to provide access to InMPs outside of clinical trials. Indeed, regulatory bodies cannot force manufacturers along this path. Potential barriers include financial and operational burdens for the manufacturer (high drug costs and limited supplies, requirement for certified expert treatment centers, need for close and extensive patient monitoring and follow-up, administrative efforts). Manufacturers may also worry that poor patient outcomes or adverse events arising during expanded access treatment may jeopardize any subsequent new drug application (6). In reality, the latter fear appears unfounded. Over 10-years, only two out of 11,000 EAP requests to the FDA resulted in a clinical hold being placed on the development of a commercial drug due to adverse events, and in both cases, the development continued after the issues were addressed (24, 25).

The main purpose of EAP is to provide patients with early access to InMPs. Nevertheless, data from trials with integrated EAP, while not comparable to that generated in randomized controlled trials, can provide valuable information that is relevant to a new drug application. This is particularly the case in rare cancers and other disease where Phase III data may be limited (26). The provision of InMPs for EAP with a detailed protocol for guidance and advice on safety precautions, allows patients access to an InMP until marketing authorization is available in that country. It also provides informative safety and efficacy data to both manufacturers and the scientific and regulatory organizations outside the setting of a formal clinical trial (27). EAP data from patients who have not participated in clinical trials have also been used by regulatory agencies as part of the approval process. For example, while a randomized controlled trial provided the primary safety and efficacy data for the FDA’s approval of lutetium Lu 177 dotatate, a radioactive drug for gastroenteropancreatic neuroendocrine tumors, approval also relied on safety and efficacy data from a single-arm study based on EAP (28).

Another example is that of blinatumomab. This bispecific T-cell engager (BITE), which enables a patient’s T-cells to recognize malignant B-cells, was originally approved for the treatment of adults with relapsed or refractory B-precursor acute lymphoblastic leukemia. It subsequently received a supplementary indication approval in the pediatric population, supported in part by data from 41 children under the age of 18 in a single-arm, open-label, EAP protocol. Indeed, as most InMPs are studied on adults first, EAP might be particularly beneficial for pediatric populations, who might otherwise face substantial delays before treatments for life-threatening rare cancers and diseases become available. A retrospective analysis of all single patient IND applications from a single pediatric oncology institution over a 2-year period reported that the FDA approved all 171 submitted requests; lack of a pediatric clinical trial (65%) was the most common reason for EAP application (29).

Manufacturers should therefore plan for EAP early in the life cycle of an InMP so that they can be endorsed as soon as there is sufficient evidence to suggest a positive benefit-risk profile and/or a well understood safety profile to allow for informed use of the product in a patient. In this manner, real-world data can be collected from expanded access participants and used as evidence of efficacy and safety during the regulatory approval process. This can be optimized through the grouping of patients into relevant cohorts, the establishment of patient registries, and increased collaboration with regulatory bodies to determine the structure of expanded access programs (30). The collection of real-world data also meets the value-based healthcare requirements increasingly demanded by healthcare authorities. In the oncology field, several organizations have developed frameworks to assess value including the European Society for Clinical Oncology (ESMO) Value Framework (31), the National Comprehensive Cancer Network (NCCN) Evidence Blocks (32), and the American Society of Clinical Oncology (ASCO) Value Framework (33). Clinical trials with integrated EAP may provide supplemental data on health outcomes defined by these frameworks including survival benefits, quality of life, symptomatic relief, and avoidance of toxicity.

Few studies provide information on physician perspectives concerning EAP (34−37). Those available indicate that applying for access to medications in development is poorly understood, which presents a barrier to obtaining investigational InMPs. Commonly reported concerns are lack of safety and oversight; reimbursement; the amount of time and effort required for the application; unfamiliarity with the regulatory process; provisions for data collection; and potential heightening of patient expectations (34, 35). One study of pediatric oncologists found that providers from larger institutions or with more than 15 years of clinical experience were more likely to complete an application and obtain investigational agents for their patients (36).

Our survey has also identified a clear educational need surrounding EAP processes and regulatory requirements, roles and responsibilities, and data collection. Physicians need to be informed about EAP and other preapproval pathways so that they can address questions from patients interested in following this pathway. However, there are few if any training resources available (36–38). Greater clarity is required on the application process for EAP set in place by pharmaceutical companies, and country-specific regulations and processes for EAP applications. Previous data have shown a significant association between the clarity of the application process for an EAP set in place by the pharmaceutical company and the number of applications submitted (38). However, the onus should not be solely on the sponsor. Pharmaceutical companies, regulatory authorities and healthcare and patient bodies all need to collaborate to provide educational models that explain the different types of EAP programs available, country and/or regional EAP regulations, and the application process. This joint effort should include information on: the different types of EAP (e.g. individual patient vs investigational new drug request for a group of patients, for example to bridge the gap between completion of successful Phase III trials and drug approval); a clear description of the application process; precision on the roles and responsibilities of treating physicians, pharmacists, and manufacturers for individual vs midsize/group/cohort EAP; and the importance of safety data collection and obligations for reporting serious adverse events to the respective competent authorities. There is also an obligation to support the patient and their family with information and education so that they have a clear understanding of the nature of an EAP and the potential benefits and risks involved. A recent initiative by the FDA has brought together patient advocacy organizations, the pharmaceutical industry, and the federal government to provide a one-stop resource for single-patient EAP under the Expanded Access Navigator service (39).

Information is also required on who should pay for access to InMPs, and how much they should pay? In most countries, treatments offered under EAP cannot be priced for profit, but costs remain unaffordable for most patients and medical insurance policies do not generally cover treatments that have not been approved by regulators. Some EU Member States have nationalized programs in place that allow companies to access markets through the donation of medication for at-risk groups of patients. Examples include the German and Italian Compassionate Use Programs and the UK Early Access to Medicines Scheme. If an InMP is approved for one of these programs, the countries health service is obligated to pay for people who fit the criteria to have access to the treatment; these initiatives carry no revenue for the donating organization. Other schemes in Europe allow the pharmaceutical industry to access a market pre-authorization and derive revenue from a product, albeit for a short period of time. An example is the French Temporary Utilisation Program (Autorisation Temporaire d’Utilisation, ATU), which can be used for individuals or cohorts of patients and allows the manufacturer to set the price of the product freely for 1 year, with any variation in the price paid back if there is a difference post-launch (40, 41).

In practical terms, the greater implementation of EAP protocols will also require harmonization of expanded access definitions between regulatory authorities. In European countries, there should also be compulsory adoption of EMA regulations, whereas currently they are only optional.

Competent authorities should encourage EAP applications at a country level, particularly in oncology and in rare diseases, at the same time as simplifying the application process in terms of the documents required. The COVID-19 pandemic has also highlighted the need to optimize and simplify regulatory requirements, as well as the application process and follow-up procedures to facilitate access under remote working conditions. Physicians could be educated on availability and processes for accessing EAPs through webinars and continuing medical education *via* digital, interactive platforms able to address questions in real time and 24/7. Consensus recommendations should be developed incorporating feedback from a variety of stakeholders including physicians, hospital pharmacies, manufacturing companies, regulatory bodies, insurance companies, and patient advocacy groups to provide simple, transparent, and consistent guidelines for the expanded access process. InMPs for diseases with a high unmet need, particularly if first in class, lack price benchmarks and comparators which can result in lengthy price negotiations. Recommendations should therefore include consideration of the price/cost of the InMP, decoupled from the price/reimbursement strategy after marketing authorization, similar to the ATU status in France, to support manufacturers providing the InMPs and to prevent major delays in commercialization (42).

## Conclusions

EAP programs allow InMP access to patients who might otherwise have no further treatment options available to them at the same time as meeting ethical requirements to allow patients the opportunity to receive treatment in a safe environment. EAP prograrms can also provide potential benefits for pharmaceutical companies by capturing important and usable efficacy and safety data. Efforts are needed to ensure wider implementation of trials with integrated EAP to ensure they become a key component in the development and launch process of an InMP. Our survey has identified several areas where physicians require more education to improve their knowledge and understanding of the EAP process, including those related to the application process, regulatory framework follow-up, and roles and responsibilities of those involved. Competent authorities should also consider how to optimize and simplify procedures to ensure continued use of EAP under remote working conditions such as during the COVID-19 pandemic, and invest in rapidly growing technological advancements and digitalization.

## Data availability statement

The original contributions presented in the study are included in the article/**Supplementary Material**. Further inquiries can be directed to the corresponding author.

## Ethics statement

Since no patients/healthy volunteers were involved in the survey, the ethical review and approval, along with written informed consent form was not required for the study on human participants in accordance with the local legislation and institutional requirements.

## Author contributions

AK developed the concept of the survey and manuscript outline. AK, SS, YG-M and MZ contributed equally in the development, review and approval of the manuscript. All authors contributed to the article and approved the submitted version.

## Conflict of interest

AK, is employed by Apogenix AG, Germany. SS, is employed by VMLY&R Health. YG-M, is employed by VMLY&R Health, UK. MZ, is employed by Sandoz GmbH, Germany.

## Publisher’s note

All claims expressed in this article are solely those of the authors and do not necessarily represent those of their affiliated organizations, or those of the publisher, the editors and the reviewers. Any product that may be evaluated in this article, or claim that may be made by its manufacturer, is not guaranteed or endorsed by the publisher.

## Supplementary material

Supplementary Figure 1 Evidence considered highly to very highly important to support a decision to request innovative medicinal products under early access provision.

Supplementary Figure 2 Rating of supporting evidences when requesting innovative medicinal product under early access provision: **(A)** respondents from Europe, **(B)** respondents from USA.

Supplementary Figure 3 What typical challenges do you face when dealing with early access provision?

Supplementary Figure 4 Comparison of challenges faced by European and US respondents when dealing with early access provision.
