# Supplementary material for: Early access provision: Awareness, educational needs and opportunities to improve oncology patients’ access to care
Source: Front Oncol. 2022 Oct 26;12:714516. doi: 10.3389/fonc.2022.714516 (PMC9643861; doi:10.3389/fonc.2022.714516)
Supplement: Supplementary Figure 1 — Evidence considered highly to very highly important to support a decision to request innovative medicinal products under early access provision. [file Presentation_1.pptx]

## Slide 1
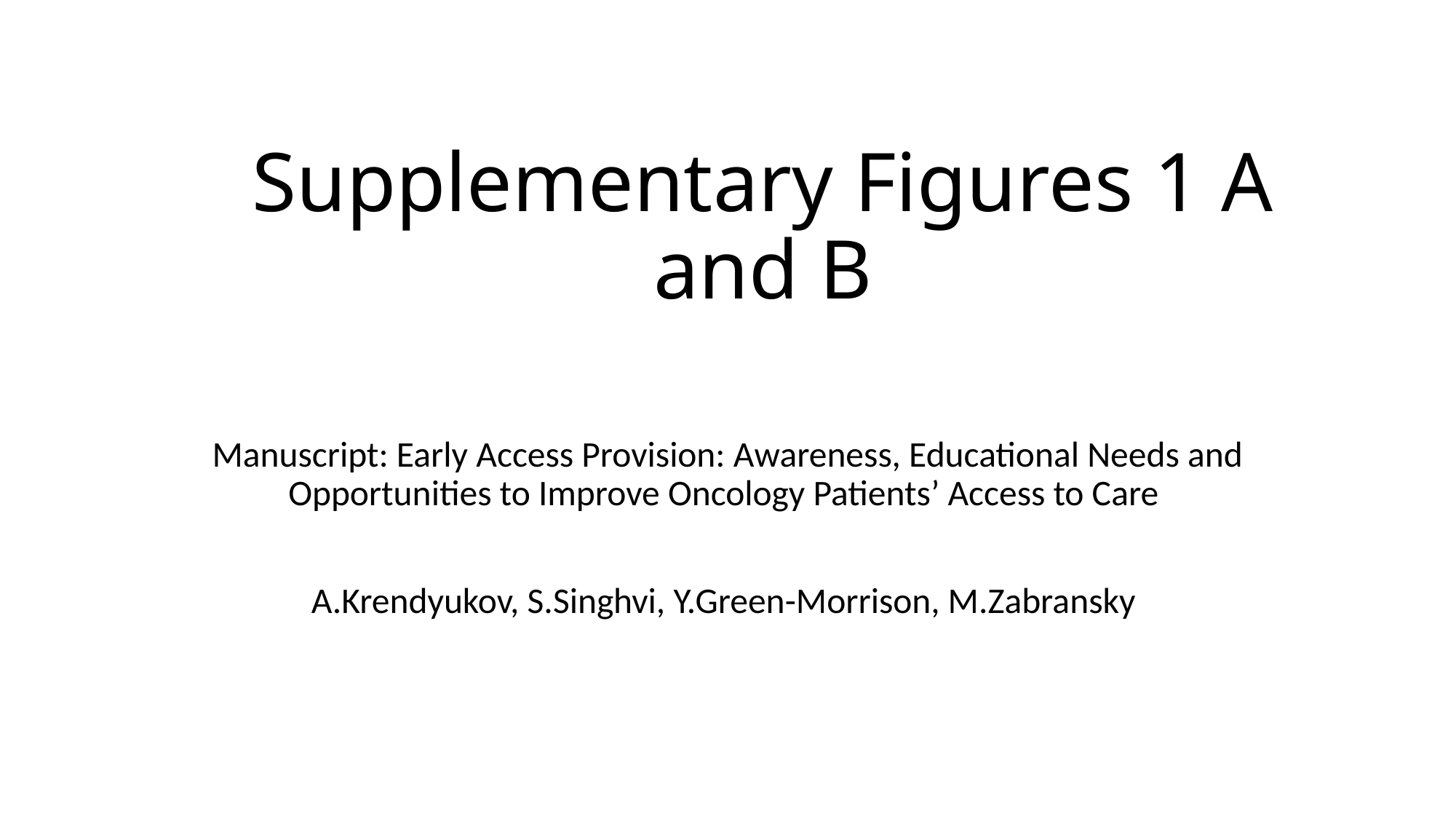

# Supplementary Figures 1 A and B
Manuscript: Early Access Provision: Awareness, Educational Needs and Opportunities to Improve Oncology Patients’ Access to Care
A.Krendyukov, S.Singhvi, Y.Green-Morrison, M.Zabransky

## Slide 2
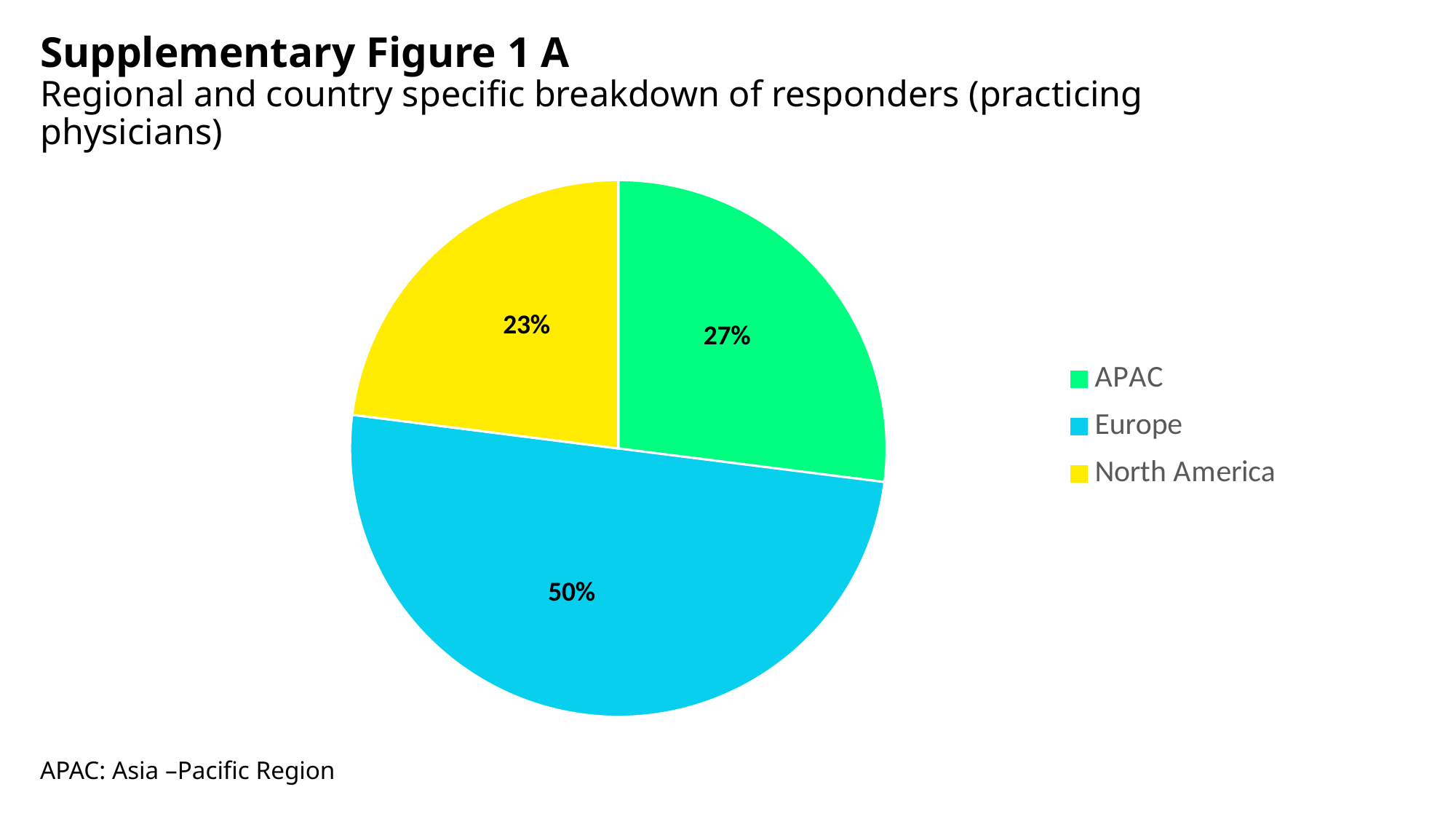

# Supplementary Figure 1 A Regional and country specific breakdown of responders (practicing physicians)
### Chart
| Category | |
|---|---|
| APAC | 0.27 |
| Europe | 0.5 |
| North America | 0.23 |APAC: Asia –Pacific Region

## Slide 3
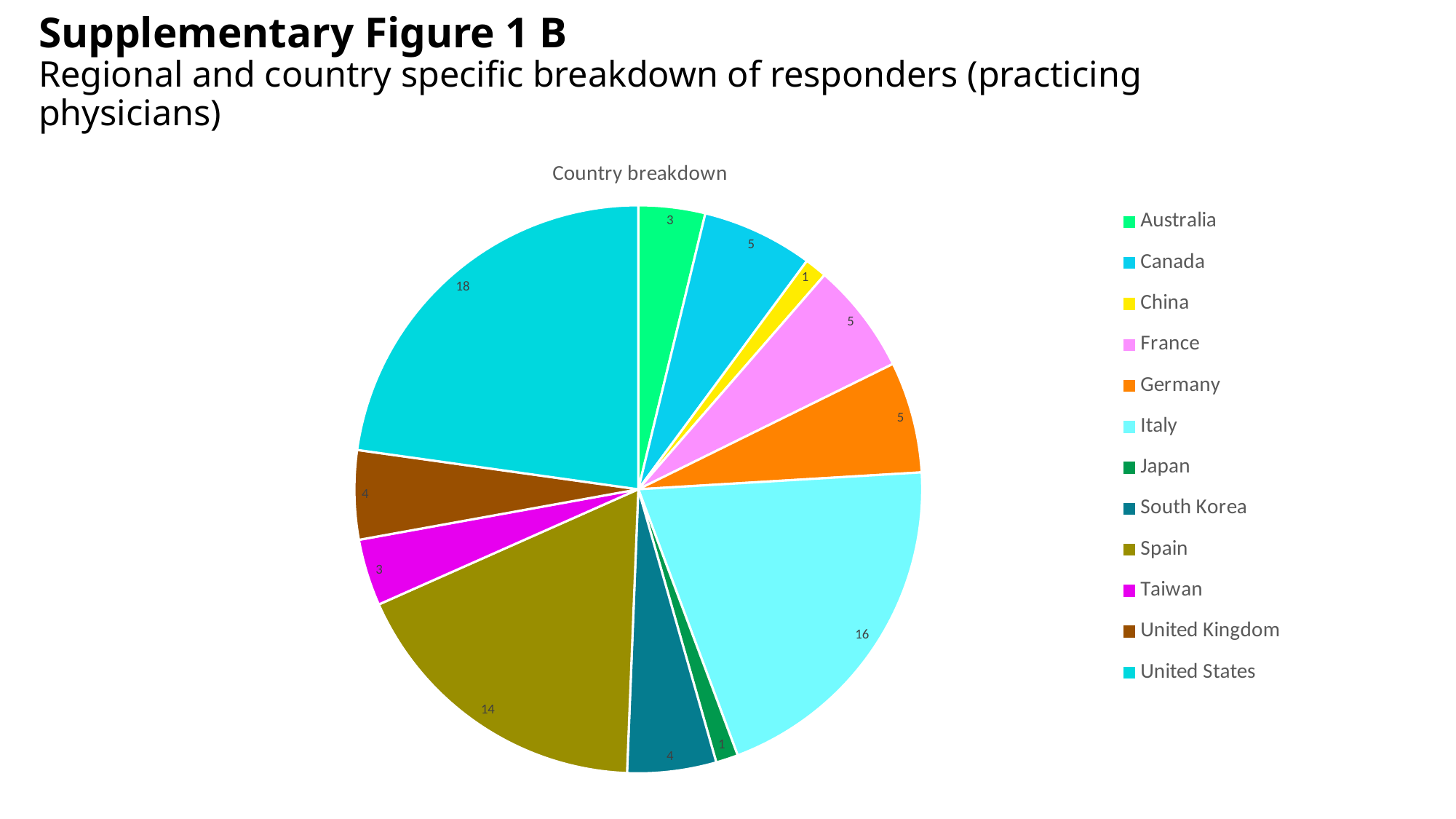

# Supplementary Figure 1 B Regional and country specific breakdown of responders (practicing physicians)
### Chart: Country breakdown
| Category | |
|---|---|
| Australia | 3.0 |
| Canada | 5.0 |
| China | 1.0 |
| France | 5.0 |
| Germany | 5.0 |
| Italy | 16.0 |
| Japan | 1.0 |
| South Korea | 4.0 |
| Spain | 14.0 |
| Taiwan | 3.0 |
| United Kingdom | 4.0 |
| United States | 18.0 |
