# Supplementary material for: Early access provision: Awareness, educational needs and opportunities to improve oncology patients’ access to care
Source: Front Oncol. 2022 Oct 26;12:714516. doi: 10.3389/fonc.2022.714516 (PMC9643861; doi:10.3389/fonc.2022.714516)
Supplement: Supplementary Figure 2 — Rating of supporting evidences when requesting innovative medicinal product under early access provision: (A) respondents from Europe, (B) respondents from USA. [file Presentation_2.pptx]

## Slide 1
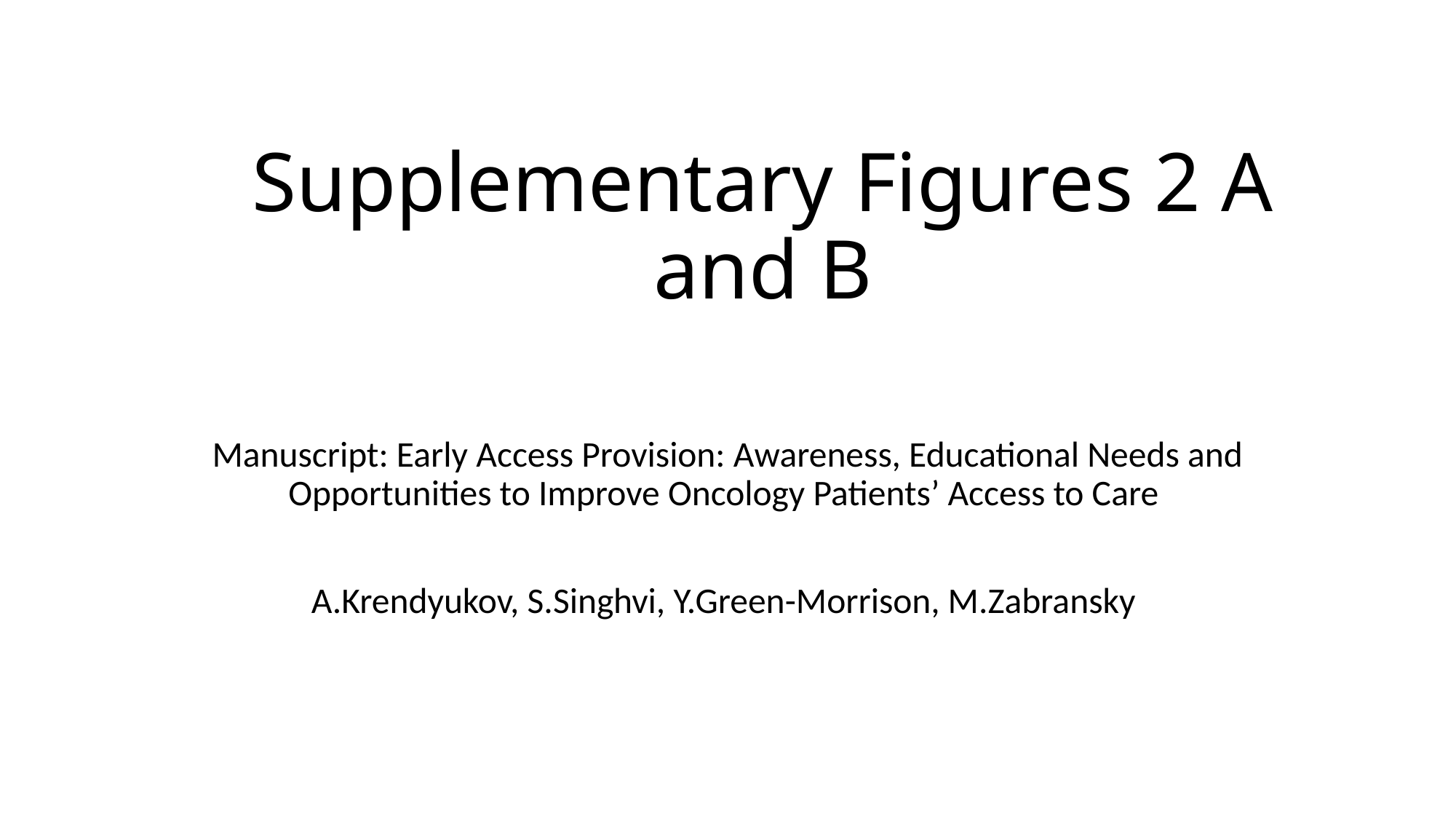

# Supplementary Figures 2 A and B
Manuscript: Early Access Provision: Awareness, Educational Needs and Opportunities to Improve Oncology Patients’ Access to Care
A.Krendyukov, S.Singhvi, Y.Green-Morrison, M.Zabransky

## Slide 2
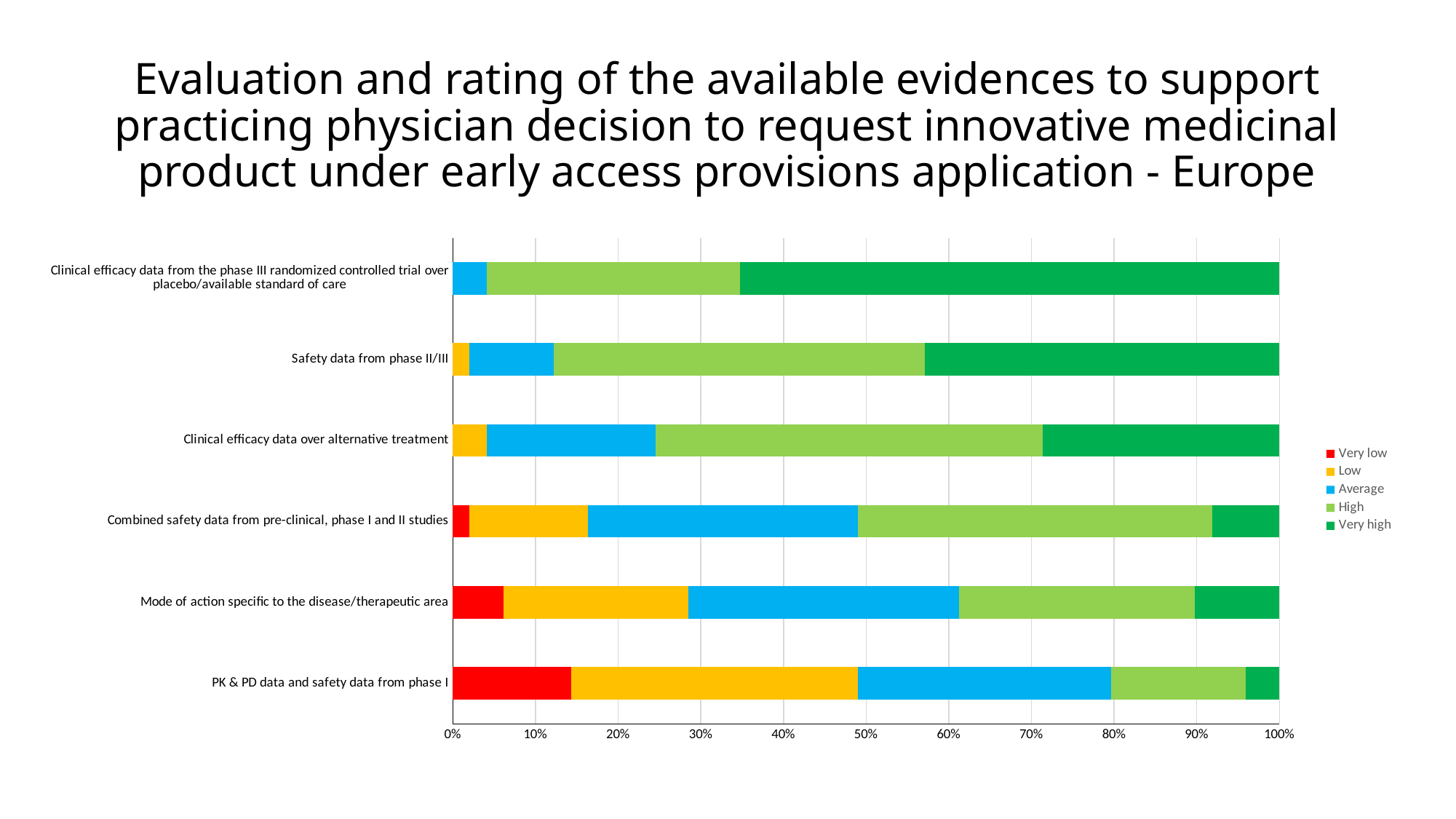

Evaluation and rating of the available evidences to support practicing physician decision to request innovative medicinal product under early access provisions application - Europe
### Chart
| Category | Very low | Low | Average | High | Very high |
|---|---|---|---|---|---|
| PK & PD data and safety data from phase I | 0.143 | 0.347 | 0.306 | 0.163 | 0.041 |
| Mode of action specific to the disease/therapeutic area | 0.061 | 0.224 | 0.327 | 0.286 | 0.102 |
| Combined safety data from pre-clinical, phase I and II studies | 0.02 | 0.143 | 0.327 | 0.429 | 0.082 |
| Clinical efficacy data over alternative treatment | 0.0 | 0.041 | 0.204 | 0.469 | 0.286 |
| Safety data from phase II/III | 0.0 | 0.02 | 0.102 | 0.449 | 0.429 |
| Clinical efficacy data from the phase III randomized controlled trial over placebo/available standard of care | 0.0 | 0.0 | 0.041 | 0.306 | 0.653 |

## Slide 3
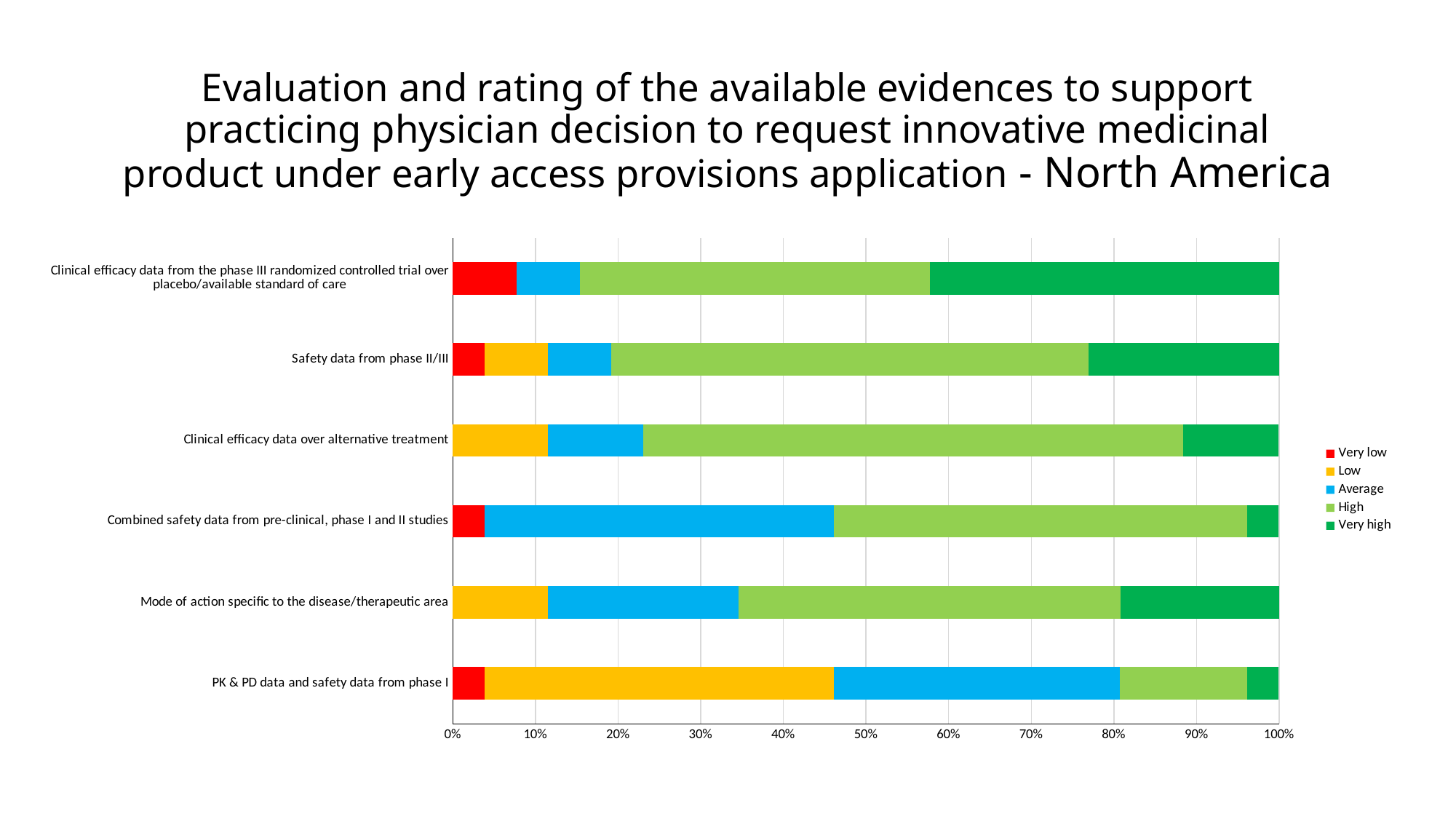

Evaluation and rating of the available evidences to support practicing physician decision to request innovative medicinal product under early access provisions application - North America
### Chart
| Category | Very low | Low | Average | High | Very high |
|---|---|---|---|---|---|
| PK & PD data and safety data from phase I | 0.038 | 0.423 | 0.346 | 0.154 | 0.038 |
| Mode of action specific to the disease/therapeutic area | 0.0 | 0.115 | 0.231 | 0.462 | 0.192 |
| Combined safety data from pre-clinical, phase I and II studies | 0.038 | 0.0 | 0.423 | 0.5 | 0.038 |
| Clinical efficacy data over alternative treatment | 0.0 | 0.115 | 0.115 | 0.654 | 0.115 |
| Safety data from phase II/III | 0.038 | 0.077 | 0.077 | 0.577 | 0.231 |
| Clinical efficacy data from the phase III randomized controlled trial over placebo/available standard of care | 0.077 | 0.0 | 0.077 | 0.423 | 0.423 |
