# Supplementary material for: Early access provision: Awareness, educational needs and opportunities to improve oncology patients’ access to care
Source: Front Oncol. 2022 Oct 26;12:714516. doi: 10.3389/fonc.2022.714516 (PMC9643861; doi:10.3389/fonc.2022.714516)
Supplement: Supplementary Figure 3 — What typical challenges do you face when dealing with early access provision? [file Presentation_3.pptx]

## Slide 1
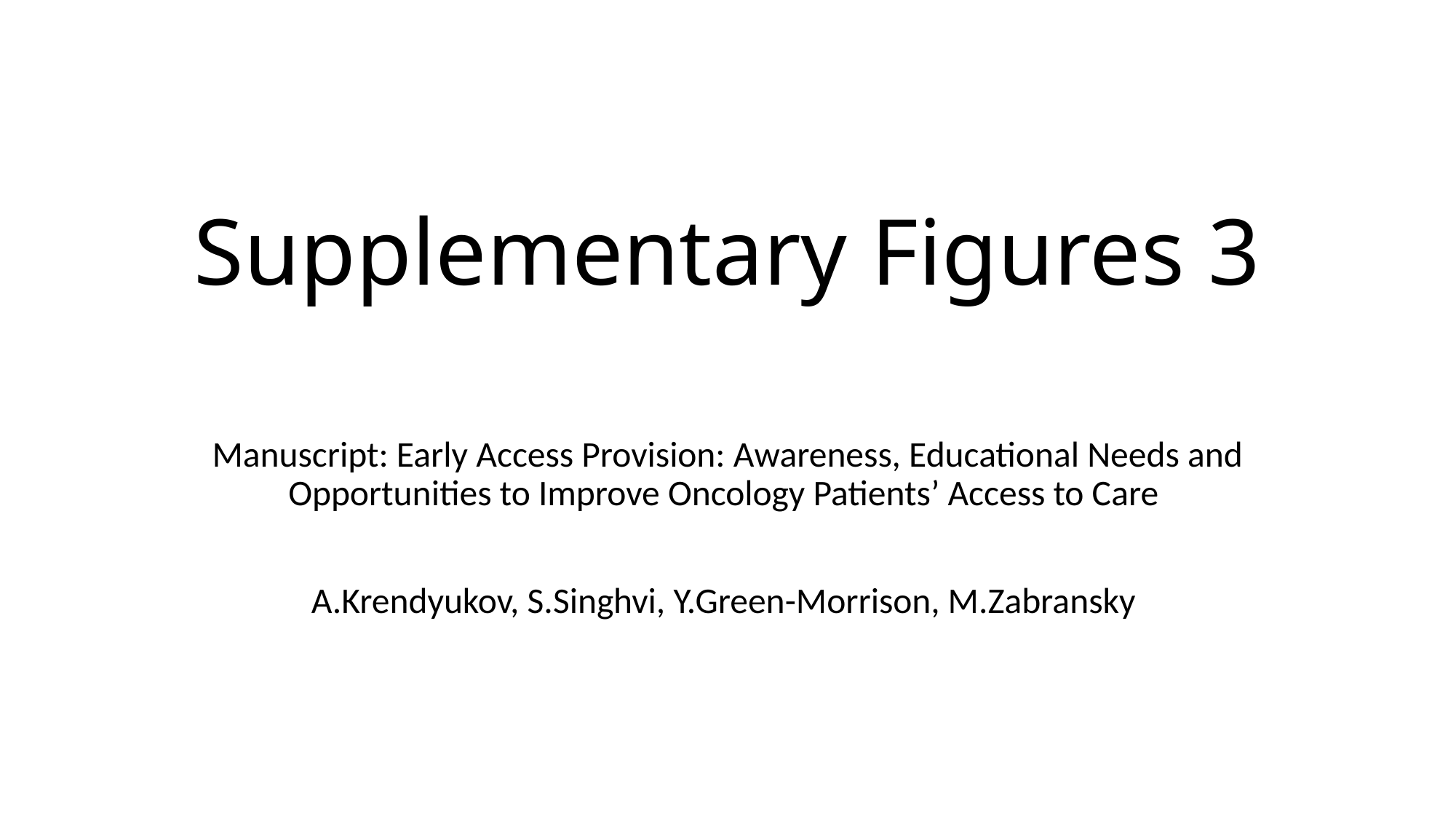

# Supplementary Figures 3
Manuscript: Early Access Provision: Awareness, Educational Needs and Opportunities to Improve Oncology Patients’ Access to Care
A.Krendyukov, S.Singhvi, Y.Green-Morrison, M.Zabransky

## Slide 2
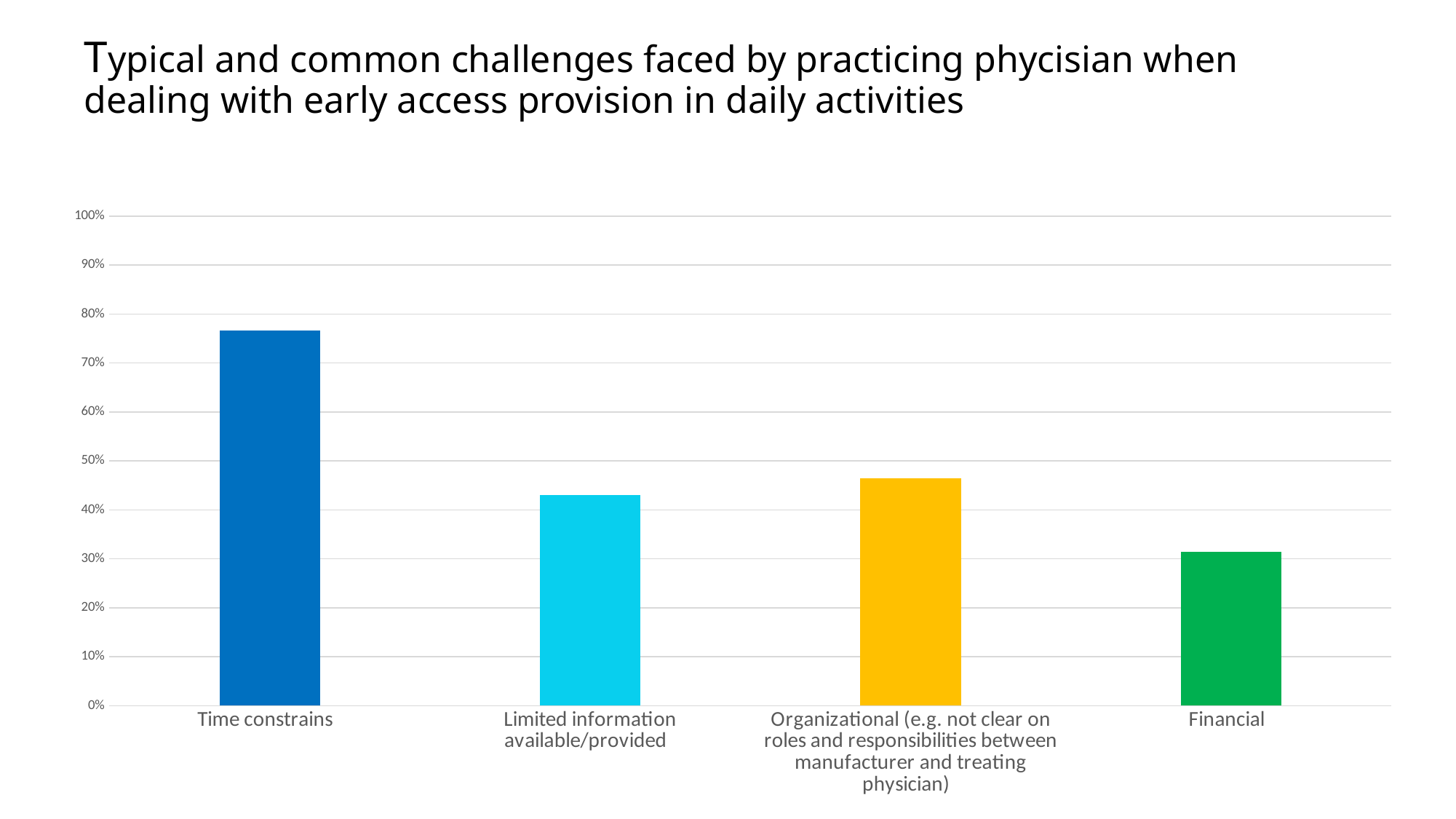

# Typical and common challenges faced by practicing phycisian when dealing with early access provision in daily activities
### Chart
| Category | |
|---|---|
| Time constrains   | 0.767 |
| Limited information available/provided   | 0.43 |
| Organizational (e.g. not clear on roles and responsibilities between manufacturer and treating physician)   | 0.465 |
| Financial   | 0.314 |
