# Supplementary material for: Early access provision: Awareness, educational needs and opportunities to improve oncology patients’ access to care
Source: Front Oncol. 2022 Oct 26;12:714516. doi: 10.3389/fonc.2022.714516 (PMC9643861; doi:10.3389/fonc.2022.714516)
Supplement: Supplementary Figure 4 — Comparison of challenges faced by European and US respondents when dealing with early access provision. [file Presentation_4.pptx]

## Slide 1
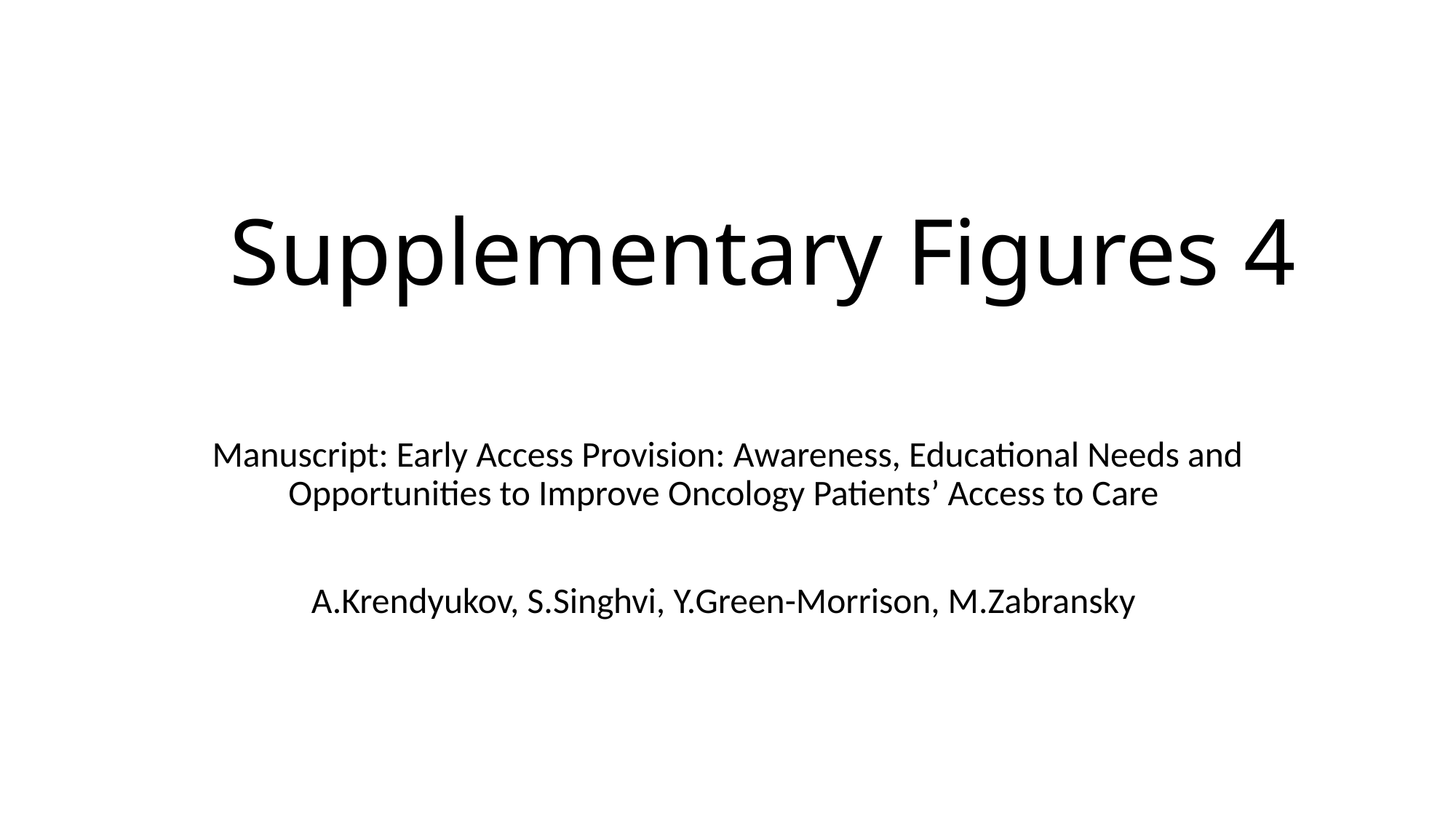

# Supplementary Figures 4
Manuscript: Early Access Provision: Awareness, Educational Needs and Opportunities to Improve Oncology Patients’ Access to Care
A.Krendyukov, S.Singhvi, Y.Green-Morrison, M.Zabransky

## Slide 2
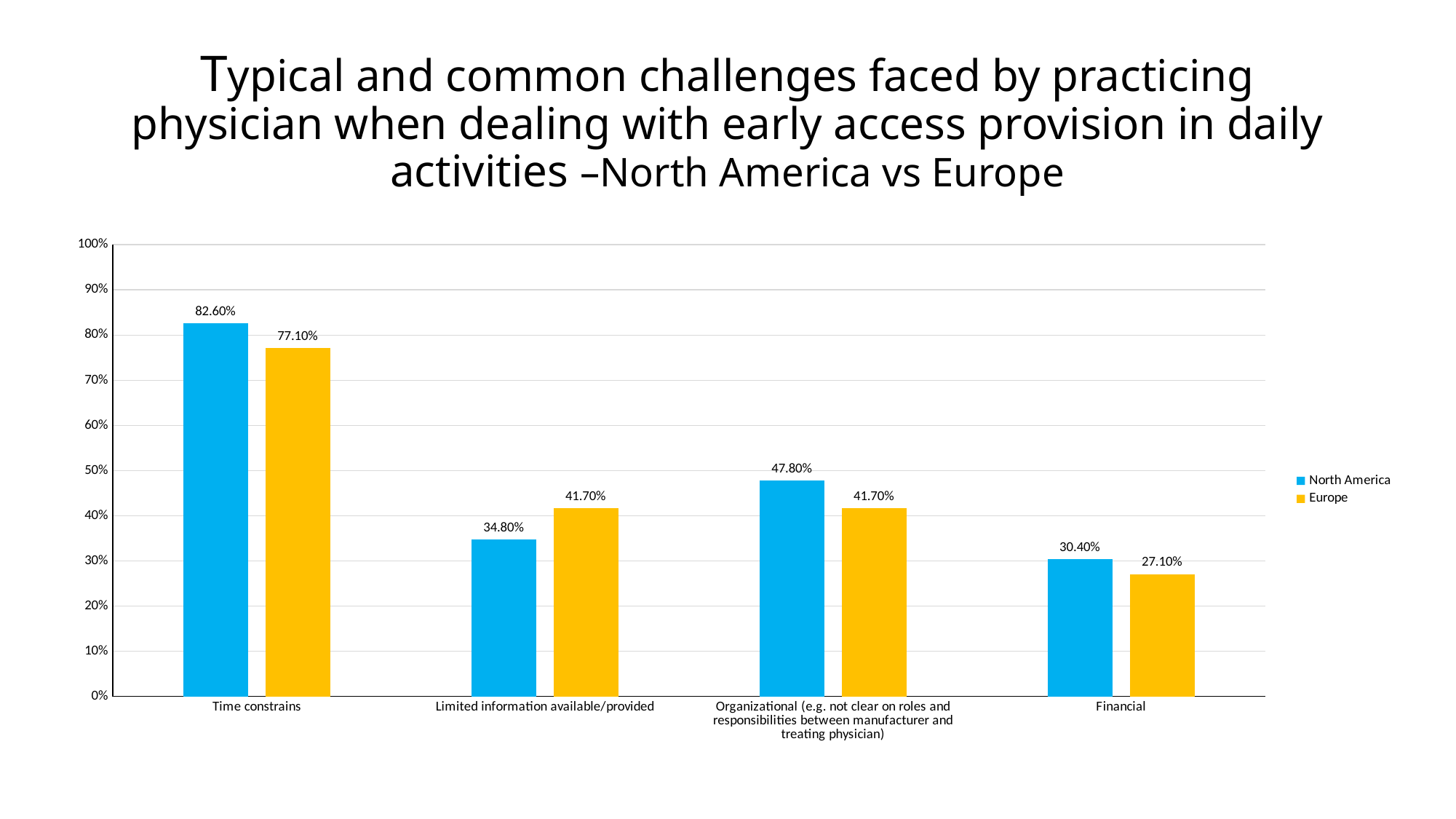

Typical and common challenges faced by practicing physician when dealing with early access provision in daily activities –North America vs Europe
### Chart
| Category | North America | Europe |
|---|---|---|
| Time constrains | 0.826 | 0.771 |
| Limited information available/provided | 0.348 | 0.417 |
| Organizational (e.g. not clear on roles and responsibilities between manufacturer and treating physician) | 0.478 | 0.417 |
| Financial | 0.304 | 0.271 |
